# Chronic Cough Revealing a Tracheal Diverticulum: A Case Report

**DOI:** 10.7759/cureus.26958

**Published:** 2022-07-18

**Authors:** Oumayma Haloui, Ouiame Nabou, Mohammed Musallam, Afaf Thouil, Hatim Kouismi

**Affiliations:** 1 Pulmonology, Centre Hospitalier Universitaire Mohammed VI, Oujda, MAR; 2 Pulmonology, Mohammed VI University Hospital, Oujda, MAR; 3 Pulmonology, Mohamed First University, Oujda, MAR; 4 Faculty of Medicine, Mohamed First University, Oujda, MAR

**Keywords:** acquired, congenital, tracheal wall, cysts, tracheal diverticulum

## Abstract

Tracheal diverticulum (TD) is a rare entity in the literature. It is the consequence of a congenital or acquired weakness of the tracheal wall. The principal difference lies in the histological characteristics of the wall. Most cases are asymptomatic, but when symptoms are found, they are usually not specific. Therefore, the diagnosis is made based on the results of CT. We report a case of a 62-year-old female presenting with a chronic cough. A diagnosis of the TD was established on the basis of a thoracic CT scan.

## Introduction

Tracheal diverticulum (TD) is an uncommon condition, generally discovered fortuitously, and characterized by single or several tracheal wall evaginations [1]. TD was first reported by Rokitansky in 1838 [2]. The estimated global prevalence is about 1%, based on a series of autopsies by MacKinnon D [3]. It is a benign entity, but it may induce certain chronic symptoms (e.g., cough) and seldom airway obstruction [4]. TD can be either congenital or acquired [5]. We report a case of a female patient with chronic cough who was diagnosed with TD.

## Case presentation

We report a case of a 62-year-old female patient with a past medical history of hyperthyroidism on carbimazole and propranolol and type 2 diabetes on insulin. The patient complained of a chronic dry cough with no signs of hemoptysis, dyspnea, fever, or dysphagia and no signs of deterioration in general condition for one year. In addition, the physical examination revealed no abnormalities, including pulmonary auscultation.

**Figure 1:**
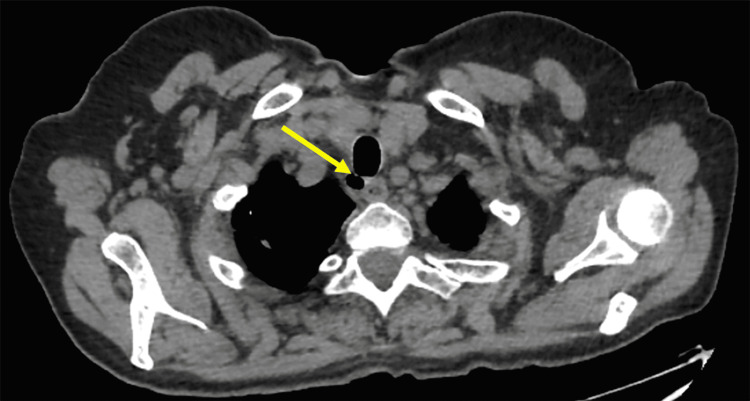
CT scan of the chest showing the tracheal diverticulum manifesting as a pouch on the right posterolateral wall.

A Thoracic CT scan revealed a cystic lesion of 1 cm connected to the tracheal lumen. It was located in the right postero-lateral part of the trachea, with associated bilateral calcified micronodular and nodular apical lesions that looked like sequellar lesions (stable appearance on two scans performed six months apart) without emphysema or pneumomediastinum (Figure 1).

Bronchial endoscopy was entirely normal (no change in the wall of the tracheal surface). Bacteriological investigations, including the search for acid-resistant and common germs, returned negative. Spirometry was done and came up with normal results; no obstructive syndrome or restrictive pattern was found.

The diagnosis of TD was established given the postero-lateral location at the cervicothoracic junction. The decision was to go for conservative treatment as the patient remained asymptomatic since diagnosis with a spontaneous resolution of the cough. The duration of the follow-up was nine months.

## Discussion

TD is a rare type of paratracheal air cyst with an estimated prevalence of 1-4% [6,7]. Results on gender predominance are still not unified, as several studies have found that tracheal diverticulum is more widespread in women [8,9], while others have reported a higher prevalence in men [7]. Tracheal diverticula are categorized into two subtypes: the congenital and the acquired diverticulum [5]. Congenital tracheal diverticula usually occur as malformations during the development of the trachea and are usually associated with other congenital conditions. They are more described among male patients but are also known for more common structural parameters such as small diameters and frequent direct contact with the trachea. Their wall is made of cartilage, smooth muscle, and epithelium, similar to that of the trachea. Furthermore, 98% are positioned next to the vertebrae T1-T3, above the carina, or 4 to 5 cm below the vocal cords [1,5,10-12].

Acquired tracheal diverticula are due to an intraluminal pressure increment that causes a herniation of the epithelial membrane through a weak point in the tracheal wall. Unlike congenital TDs, they are larger, with wider openings to the tracheal lumen and thinner walls made exclusively of epithelium. In addition, they occur at different localizations of the trachea and are rarely multiple [1,5,10,11]. In fact, the increase in intraluminal pressure responsible for TD may be due to chronic coughing or weakened structures secondary to tracheal surgical procedures, leading to an external invagination of the mucous membrane through vulnerable points of the trachea [13,14]. It has also been hypothesized that acquired diverticulum may be induced by cystic distension and mucous gland duct thickening [3, 15].

Clinically, tracheal diverticula are usually asymptomatic and discovered fortuitously during a radiological investigation. Symptoms such as cough, dyspnea, dysphonia, vocal cord paralysis, and pain are explained by the local mass effect of the diverticulum [6, 16]. Additionally, repeated infections and chronic manifestations, including chronic cough, dyspnea, hemoptysis, or stridor, are favored by the tracheal diverticulum as it can be a reservoir for secretions stagnation [6, 17].

A thin-section CT scan of the trachea coupled with a three-dimensional reconstruction illustrates best the communication between the cyst and the airway. It is considered the best diagnostic approach for the tracheal diverticulum [6, 18]. Furthermore, the appearance of tracheal diverticula depends on their size and content. According to the literature, most tracheal diverticula are located in the right posterolateral wall of the trachea, leaving the diverticula on the left side extremely rare [1, 6, 19]. Additionally, a CT scan may be performed to distinguish between the acquired and congenital forms according to some structural variations, such as the presence of cartilage and the size of the neck of the diverticulum [20]. Bronchoscopy can be useful in locating the opening, although the diverticulum with a narrow opening may be missed [1,20]. Indeed, in the available data, tracheal diverticula are usually described as having an ovoid pedunculated shape with a large vertical axis, where most communications may not be detected due to their small size even during bronchoscopy [1,3,6]. Several differentials are known, including laryngocele, pharyngocele, Zenker's diverticulum, apical hernia of the lung, apical bullae, and pneumomediastinum [1,6,21].

The choice of treatment is decided according to several factors such as age, physical condition, and the symptoms presented [6]. Conservative treatment is recommended for elderly subjects [22] and especially for asymptomatic diverticula [17]. In fact, conservative treatment includes antibiotics, mucolytic agents, bronchodilators, and physiotherapy [6, 16]. Surgical resection is the most appropriate treatment for a symptomatic diverticulum [23]. Surgery may be necessary for patients in whom conservative treatment has failed. It is usually preferred in young symptomatic patients [24]. Intubation should be performed carefully for these patients because the defect may lead to ventilatory failure due to mispositioning of the endotracheal tube [2, 6]. The approach varies based on the position of the diverticulum [6]. A lateral cervical approach may be taken to avoid thoracotomy [6]. Other alternatives include endoscopic laser or electrocoagulation cautery and fulguration with potentially lower risk than excision [6, 16].

## Conclusions

TD is a rare clinical disease. It can be either acquired or congenital. Its diagnosis is based essentially on thoracic high-resolution computed tomography (HRCT) and is most often asymptomatic. The indication of conservative or surgical treatment with resection of the diverticulum depends on several factors. However, resection of the diverticulum is the proper surgery and should be necessary for symptomatic patients or in whom conservative treatment has failed. Therefore definite classification prior to surgery is mandatory (acquired or congenital diverticulum). Risks of surgery include damage to the recurrent laryngeal and esophageal nerves.
